# Supplementary material for: Are carbon emissions trading and green financial instruments synergistic? -Comprehensive quantitative research based on content analysis
Source: PLoS One. 2024 Mar 7;19(3):e0298601. doi: 10.1371/journal.pone.0298601 (PMC10919601; doi:10.1371/journal.pone.0298601)
Supplement: S1 Data — (ZIP) [file pone.0298601.s001.zip › Data/地级市Z_F工作报告.zip!/╕ú╜¿/╞╬╠∩╩╨/└┤╫╘╣1⁄2╓┌║┼í╛┬φ┐╦╩2╛▌═°í┐_2006.docx]

莆田市人民政府市长 詹 毅

各位代表：

现在，我代表莆田市人民政府向大会作工作报告，请各位代表连同《莆田市国民经济和社会发展第十一个五年规划纲要（草案）》一并审议，并请政协委员和其他列席人员提出意见。

一、2005年工作回顾和“十五”计划目标完成情况

2005年，全市上下坚持以科学发展观为指导，按照建设海峡西岸经济区的总体部署，加快建设“产业集聚强劲、配套功能完善、生态环境优美”的湄洲湾港口城市，经济社会取得长足进步，形成速度、结构、效益较为协调的良好发展局面。

国民经济快速增长。农业在结构调整中稳步发展，农林牧渔业总产值84.14亿元，增长6.2%；工业高速增长，规模以上工业产值376.37亿元，增长32.8%，增幅居全省第二位。工业经济效益大幅提高，规模以上工业综合效益指数为151.5，同比提高10.1个百分点，全年实现利润14.28亿元，增长20.6%；投资拉动作用凸显，全社会固定资产投资额115.41亿元，增长47.3%，增幅居全省第一位；消费水平不断提高，社会消费品零售总额128.17亿元，增长11.7%；外贸出口总额11.17亿美元，增长10.9%。这些因素有效支撑经济的快速增长，按照经济普查后新核算方法，初步核算全市生产总值359.93亿元，增长15.3%，增幅居全省第二位；财政总收入29.46亿元，可比增长18.2%，其中地方级财政收入14.56亿元，可比增长26.6%，增幅居全省第一位。经济快速发展带动城乡居民收入稳步增加，城镇居民人均可支配收入11026元，增长7.8%，农民人均纯收入4486元，增长9.6%，增幅居全省第二位；全年城镇登记失业率控制在2.49%以内。实施品牌战略，雪津、金威世家两件商标获中国驰名商标认定，中国驰名商标增至3项；4项产品获得2005年中国名牌产品称号，中国名牌产品增至6项，居全省第三位；5项产品获2005年国家免检产品，国家免检产品增至14项，居全省第二位。

项目带动成效明显。全市59个在建重点项目完成投资47.6亿元，固定资产投资中重点建设项目的比重、重点建设项目中工业项目的比重比上年分别提高12个和18个百分点。一批具有战略性意义的重大项目取得新进展，总投资30亿美元的林浆纸一体化项目正式签约；福厦铁路木兰溪特大桥控制性工程已动工；向莆铁路项目列入国家“十一五”规划，建设方案已通过铁道部评审；湄洲湾港口铁路支线，铁道部已同意委托向莆铁路的设计单位一并设计，争取同步设计、同步建设、同步投入使用；省道201线三个路段开工建设；木兰溪防洪二期工程、澄峰围垦工程顺利推进，金钟水利枢纽工程可研报告已通过国家水利部审查，正上报国家发改委批复，并获得中国农业银行总行贷款承诺；LNG气电一体化项目的站线工程正式开工建设，莆田燃气电厂已获国家发改委正式核准，三通一平工作基本完成；国家级进口木材检疫除害处理区完成一期建设，并通过国家正式考核验收。中原港务30万吨中心油库、莆田风力发电场和仙游抽水蓄能电站等项目前期工作取得突破；滨海路堤等一批重大项目正在抓紧规划。园区效应进一步显现，新增工业项目75个，完成固定资产投资20.82亿元，增长53.4%；企业总产值达113.81亿元，增长37.8%。

加大招商引资力度，民营经济迅速壮大。全力实施民资回归工程，先后举办了温州和香港经贸活动周，举行了秀屿国家级木材加工区招商会、东庄医疗医药展销会、南京、昆明等地投资项目推介会，参加了“6·18”项目成果对接交易会和“9·8”投洽会，组团赴日本、美国开展木材产业招商推介活动，成功举办了第二届世界莆商大会、第四届世界兴安同乡恳亲会和第七届妈祖文化旅游节，形成民营资本踊跃回归的喜人局面。全年合同利用外资4.64亿美元（历史可比口径），增长75.32%，实际利用外资3.2亿美元，增长25.65%；新增投资项目609个，计划投资总额277.3亿元。

城乡面貌继续改观。完成城市总体规划修编《纲要》及湄洲湾港口新城发展规划方案，组织编制凤凰山风景区、妈祖城总体规划。完成城市基础设施建设投资3.52亿元，动工建设荔园路二期、疏港路（工业大道），续建荔园路一期、荔涵大道城厢段和荔城段、东圳东路、延寿北路、东园路、涵江人民街。续建了东岩山公园、延寿公园，建成天九湾广场。城市污水处理率达45.8%，建成区绿化覆盖率达36.8%，人均公共绿地面积达6.04平方米，自来水普及率达96.6%。不断巩固农村税费改革成果，继续深化集体林权制度改革，全面落实支农惠农政策。推进农村基础设施建设，水利“六千”工程年度任务全面完成，建成行政村通水工程180个，完成农村水泥公路建设560公里。着力整治农村环境卫生，农村人居环境不断改善。

投资环境不断改善。深化行政管理体制改革，强化市招投标市场管理委员会办公室和行政服务中心、投资项目服务中心、招投标中心等“一办三中心”的职能，市行政服务中心共受理申请事项64806件，办结63821件，办结率98.4%。加快“数字莆田”建设，深化机关效能建设和行风评议工作，不断优化投资环境。加大调控力度，缓解要素制约。召开莆田市重点项目融资推介会，签约27个融资项目，计划融资27.5亿元；与国家开发银行正式签订了《开发性金融合作协议》，有28个项目进入开发银行授信贷款计划，授信额度34亿元。争取到省在建重点项目农转用指标16000亩，一般工业项目农转用指标6000亩，加大征地拆迁力度，完成征地4285亩，征地率达57.7%。加大了引进外来务工人员力度，共引进1.65万人，为企业输送劳动力5.59万人，全市劳动力求人倍率从上年底的1.5降为1.36。

社会事业持续进步。大力实施科教兴市战略，国家科技进步考核排名全省第三，首获全国科技进步先进城市称号。坚持“两基”巩固提高与教育“双高普九”并重，高考录取人数增长18.5%，莆田二中、四中顺利完成新校搬迁，农村中小学危房改造顺利推进。市第五医院完成烈急性传染病房和综合楼建设，市急救中心、疾控中心即将竣工。加快建设城乡卫生服务体系，新型农村合作医疗试点工作顺利开展。市长环保目标责任制年度任务基本完成。人口自然增长率控制在6.7‰。文化体育、新闻出版、广播电视等社会事业取得新进步，外事侨务、民政、民族宗教、档案、气象、防震减灾取得新成绩，妇女、儿童、老龄、残疾人等事业有了新发展。

民主法制得到加强。自觉接受人大及其常委会监督，重视和支持人民政协履行职能。全年共办理市人大代表议案建议131件、市政协委员提案197件，满意和基本满意率达99.4%。认真落实国务院依法行政实施纲要，全面展开政府绩效评估工作，政务公开扎实推进，廉政建设和反腐败工作取得新进展。“四五”普法目标基本完成。重视群众来信来访工作，积极预防和妥善处置各类群体性事件。落实安全生产责任制，重特大安全生产事故得到有效遏制。推进“平安莆田”建设，加大打击各种违法犯罪力度，群众对社会治安状况满意及基本满意率为92.84%。

各位代表，2005年工作任务的完成，标志着“十五”目标的胜利实现，经济社会发展迈上一个新台阶。“十五”期间，全市生产总值年均增长12.7%，人均生产总值达1600美元；财政总收入和地方级财政收入分别年均增长17.9%、16.6%。经济总量和财政总收入跃居全省第七位。五年累计完成全社会固定资产投资351.26亿元，年均增长11.4%。外贸出口年均增长10.3%；五年累计完成实际利用外资13.02亿美元，年均增长2.6%；城镇居民人均可支配收入年均增长11.6%，农民人均纯收入年均增长6.6%。

产业结构在持续发展中不断优化。三次产业结构由2000年的20.0：45.3：34.7 调整为2005年的14.0：53.9：32.1，呈现出第一产业所占比重逐年下降，第二产业比重快速上升的态势。农业基础更加稳固，生产结构和产品结构更加优化，水产、水果、畜牧、蔬菜和食用菌五大农业主导产业进一步发展壮大。工业化步伐显著加快，形成了制鞋、纺织服装、电子信息、食品等一批初具规模的产业集群，能源、化工、机械制造等一批成长型产业集群，2005年工业总产值达463.69亿元。第三产业对经济发展的服务作用增强。

基础设施在突破制约中有效推进。五年间，全市新增县道168公里，形成以高速公路和国省道为主干线，辐射山区沿海的“三纵三横”交通网络；福厦铁路（莆田段）动工建设，向莆铁路、莆秀高速公路支线、湄洲岛至永春高速公路前期工作顺利推进。建成秀屿3.5万吨级多用码头及一批中小泊位，2005年港口货物吞吐量突破1000万吨。电源电网建设得到加强，新增发电能力4.2万千瓦，新增110千伏电路183公里。规划综合调控力度不断加大，改变城市建设安置模式，规范个人建房管理，住宅示范小区建设从无到有，建设了荔园小区等一批省级住宅示范小区，人均住宅面积增加12平方米。市政基础设施建设资金筹集渠道不断拓宽，五年共投入资金17.86亿元，新建和完善了荔城大道、荔园路、荔涵大道等市区路网，城市建成区扩大了8.56 平方公里，道路面积新增403万平方米，新增城市绿化覆盖面积238公顷。防灾减灾体系建设稳步推进，完成木兰溪防洪一期工程，除险加固险病水库83座，新建加固海堤47公里。铺设四级以上农村水泥路2096公里，全市行政村道路硬化率达93%。

发展后劲在项目带动中明显增强。五年间，全市累计建设了 105个重点项目，完成投资113亿元。一期工程总投资约110亿元的LNG气电一体化项目、国家级进口木材检疫除害处理区及国家级木材贸易加工示范区、佳通半钢子午胎技改工程、华港油脂和雪津啤酒10万吨扩建工程等项目正式建设；林浆纸一体化项目、中原港务30万吨中心油库、莆田风力发电场和仙游抽水蓄能电站等项目前期工作取得突破；台商投资区和闽台（莆田）海洋产业合作试验区等一批重大项目正在抓紧规划，为经济持续发展增强了后劲。

体制机制在深化改革中更具活力。实施项目带动战略和民资回归工程，推进项目持续落地和民营经济快速提升。行政区划顺利调整，理顺了体制性的障碍，推动了生产力的发展。理顺县区经济管理权限，增强县区经济活力。加快企业体制创新，国有企业改革基本完成，组建市国有资产管理机构，健全国有资产管理监督体制。出台加快工业园区建设意见，构建产业集聚平台。深化政府机构和行政审批制度改革，成立“一办三中心”，提高行政效能，促进政务公开。加快推进投融资、财税、社会事业、城市公用事业等配套改革。

社会事业在统筹兼顾中加快发展。技术创新能力不断加强，“十五”期间获得国家专利552项，比“九五”期间增长32.4 %。莆田学院、湄洲湾职业技术学院相继成立，高中阶段毛入学率达64.3%，高考录取率提高17个百分点。疾病预防控制体系和医疗救助体系基本建成，突发公共卫生事件预警及应急救治机制初步形成，顺利通过了省级卫生城市考评。市广电中心、市体育中心等一批文体设施相继建成。人口和计划生育工作得到了加强，五年间人口平均自然增长率为6.4‰。“双拥”和军民共建活动取得成效，连续两届被命名为“全国双拥模范城”。社会保障体系初步形成，环境保护和民主法制建设得到加强。

“十五”取得的成就，是在省委、省政府和市委的正确领导下，全市上下解放思想、开拓进取的结果；与各级人大及全体人民代表的依法监督，各级政协、民主党派、工商联以及人民团体的民主监督和积极参与，驻莆部队、武警官兵的大力支持，港澳台同胞、海外侨胞的支持合作分不开的。在此，我代表市人民政府，向所有关心、参与和帮助莆田经济和社会事业发展的同志们、朋友们表示崇高的敬意和衷心的感谢！

在肯定成绩的同时，我们要看到经济社会发展中存在的困难和问题：经济总量仍然较小，产业集群规模偏小，大项目还不多，比较优势还没有得到充分的发挥；资源短缺比较突出，要素市场的发育还不健全，影响发展的体制机制障碍尚未根本消除；基础设施和城市功能还不完善，城市的带动辐射能力还不强；城乡之间、县区之间发展还不平衡等等。对此，我们必须保持清醒的认识，以攻坚克难的精神加以解决。

二、努力实施“十一五”规划实现经济翻番 港城崛起

“十一五”时期是我市经济社会发展的战略转型期，也是全面推进湄洲湾港口城市在海峡西岸迅速崛起的关键时期。提请大会审议的《莆田市国民经济和社会发展第十一个五年规划纲要（草案）》（以下简称《纲要》），是根据党的十六届五中全会、省委七届十次全会精神和市委《关于制定莆田市国民经济和社会发展第十一个五年规划的建议》，集中有关部门力量，在广泛征求各方面意见的基础上制定的。《纲要》总结了“十五”计划的实施情况，分析了“十一五”的发展环境，提出今后五年经济社会发展构想、目标任务和重大项目。

“十一五”发展的总体要求是：以邓小平理论、“三个代表”重要思想和科学发展观为指导，全面落实“六个扎实”要求，坚持发展是硬道理，坚持抓好发展这个党执政兴国的第一要务，坚持以经济建设为中心，坚持用发展和改革的办法解决前进中的问题。按照“八个加快发展”、“七大关键举措”、“六个落实”和“构建九大支撑体系”的总体部署，积极推进湄洲湾港口城市建设。继续实施项目带动战略和民资回归工程，集聚要素，汇聚合力，奋力追赶，能快则快，做大经济总量，提升综合实力，抓住新机遇，立足新起点，应对新挑战，实现新跨越，为提前实现全面建设小康社会，率先基本实现现代化而努力奋斗。

“十一五”时期经济社会发展的预期目标是：在优化结构、提高效益和降低消耗的基础上，实现经济翻番、港城崛起。2010年全市生产总值突破800亿元，力争1000亿元，年均经济增长速度超过15%，人均生产总值达到全省平均水平；全社会固定资产投资额五年累计突破1000亿元，年平均投资率在30%以上；财政总收入达到70亿元，力争80亿元。人民生活明显改善，社会事业全面发展，民主法制不断加强，构建和谐社会取得新进步。

“十一五”时期经济社会发展的主要任务：一是按照“生产发展、生活宽裕、乡风文明、村容整洁、管理民主”的要求，建立以工促农、以城带乡的有效机制，扎实推进社会主义新农村建设。二是加强规划、整合湄洲湾岸线资源，重点建设十大港航项目，加快建设临海工业区和物流等港口综合配套设施，带动港口经济的繁荣。拉开城市建设框架，形成市域次中心与中心城区相呼应的城市新格局，全力推动港城崛起。三是提高产业集聚能力，力争纺织服装、机械制造、医药医疗器械、工艺美术四个产业集群工业产值各达50亿元，能源、电子信息、食品加工、化工四个产业集群工业产值各达100亿元，制鞋、林产加工两个产业集群的工业产值各达200亿元。加快发展服务业，实现二、三产业良性互动发展。建设海洋经济强市。四是稳步推进体制改革，提升对外开放水平。以改革增活力，努力在一些关键领域和重要环节取得新突破。以开放促发展，力争在引进重大项目和世界500强企业上有新的突破，把我市建成对台产业技术合作的重要基地。五是加快发展社会事业，深入实施科教兴市战略，建设创新型社会，增强自主创新能力，优先发展教育，加强人才队伍建设，完善公共卫生体系，建设文化强市。六是实施可持续发展战略，落实计划生育基本国策，大力发展循环经济，强化环境污染整治，建设资源节约型和环境友好型社会。七是构建和谐社会，重点解决好人民群众关心的就业、社会保障等问题。加强民主法制建设，大力推进依法治市进程，严格依法行政，强化社会治安综合治理，重视信访工作，落实安全生产责任制。

三、奋力攻坚 提速跃升 实现2006年开好局起好步

2006年是实施“十一五”规划的开局年。全市国民经济和社会发展的预期目标为：地区生产总值425亿元，增长15.5%；财政总收入34.5亿元，增长17.1%，其中地方级财政收入17.2亿元，增长18.1%；规模以上工业产值475亿元，增长25%；全社会固定资产投资额155亿元，增长34.3%；社会消费品零售总额增长13.5%；外贸出口总额增长16%；实际利用外资增长25%；城镇居民人均可支配收入增长7%，农民人均纯收入增长6%；城镇登记失业率控制在3.5%以内；人口自然增长率控制在7‰以内。

实现上述目标，必须做好以下几个方面工作：

（一）继续做好“三农”工作，建设社会主义新农村

扎实推进新农村建设。制定切实可行的总体规划和实施方案，按照市委提出建设社会主义新农村的十个目标，选择若干条件相对成熟的村镇开展试点工作。完善农村基础设施，铺设四级以上水泥路面300公里，重点建设仙游环山区公路、城常线、大江线等县道，建成12个陆岛交通码头和10个农村客运站，新开通一批农村客运班线。在荔城区率先实现“村村通水泥路”的基础上，城厢、涵江、秀屿三区实现“村村通水泥路”。加快两个省级村镇住宅示范小区建设。着力实施北洋水系（城区）整治工程，推进萩芦溪流域综合治理工程前期工作。抓住城市垃圾焚烧发电项目推进机会，着手全市城乡垃圾综合治理，以此作为新农村建设的一个抓手，改善城乡环境。积极推进扶贫开发和山海协作，加快发展贫困地区、海岛和老区，以及扶贫开发重点村。

大力发展现代农业。推进农业产业化，加快发展48家农业产业化经营组织，做大做强17家省级以上龙头企业，突出抓好14个市级农业标准示范区建设。认真落实扶持粮食生产政策，在稳定粮食生产的基础上，促进优势农产品向精细加工方向发展，推动和扩大农产品销售。结合林浆纸一体化项目的发展需要，建设原料林基地。加快莆台农业合作试验区建设，重点抓好秀屿前沁莆澳台农业综合开发、仙游摩天岭农业综合开发和怡园台湾水果种植等项目。加快农业高新技术的应用和推广，提升农业科技水平。加强农业基础设施建设，继续抓好水利“六千”工程和水库、海堤除险加固工程。加快自然灾害天气预警三期建设，推进石城国家一级渔港建设。

广开农民增收渠道。坚持把增加农民收入作为农业和农村工作的中心任务，继续加大农村税费改革和财政支农力度。引导发展农产品加工业、资源加工业和服务业，鼓励发展农村个体私营企业，大力发展农村经济合作组织。健全农村服务体系，加强高致病性禽流感等重大动植物疫病防控。加快发展劳务经济，积极引导和多渠道扩大农村劳动力转移，新增转移劳动力2万人。

（二）突出发展工业和提升服务业，提高产业集聚水平

全力推进工业化进程。强化工业对经济增长的支撑作用，实现工业对经济增长的贡献率达61%。实施大企业培育战略，打造一批龙头骨干企业和名牌优势产品，力争新增全市产值超亿元的工业企业10家，产值3000万元以上工业企业30家。制定和完善十大产业集群发展规划，培育工艺美术与仿古家具、林产加工、医药医疗用品等新兴产业，壮大电子信息、制鞋、纺织服装、食品加工等传统产业，扶持能源、化工、机械制造等成长产业。吸引和鼓励跨国、跨地区经营的企业在莆田设立加工基地和总部。继续实施品牌战略，争创一批中国驰名商标、中国名牌和国家免检产品。

加快发展现代服务业。整合旅游资源，推动湄洲岛滨海生态旅游区、湄屿潮音景点建设，完善九鲤湖、南少林、永兴岩等重要景区的配套设施，发展特色旅游、滨海旅游。完善城市商业网点规划，建设莆田鞋材批发市场，启动粮食物流配送中心项目，引进沃尔玛、易初莲花等大型连锁企业物流业，实现社会消费品零售总额145.5亿元。引导房地产业健康发展，加快建设一批商住项目。推进和规范小区物业管理，鼓励社会力量创办社区便民服务机构。推进交通运输、邮电通讯快速发展。规范发展律师、会计、金融、保险、评估等服务。

继续推进园区建设。推进枫亭、郊尾工业集中地规划建设，编制东峤、圣岭台商投资区规划。完善园区内水、电等基础设施和垃圾、污水及工业废弃物处理等环保设施，加快建设综合生活服务区。推进妈祖城和东吴园区堤路建设，积极申报国家级出口加工区。加快木材加工区建设，申报进口木材加工锯材出口试点。推进合众天成、天喔食品、众晶体育、金利莱服饰、信成科技、大起机械、安特二期、德基三期和台湾通讯等一批工业园区新增长点项目建设。

努力缓解要素制约。认真做好新一轮土地利用总体规划修编工作，总结征地拆迁工作经验，推进滩涂围垦和荒坡造地，积极争取计划用地指标和重点项目戴帽指标，保障重点项目用地需求。深化政银企合作，推进中小企业担保信用体系建设，落实开发银行等金融机构与我市合作协议。加强外地劳动力引进的组织和协调，以招商办法抓招工，解决好外来员工的住房，缓解劳动力紧缺。同时，加强经济运行分析监测，强化煤电油运调度。

（三）继续实施项目带动，增强经济发展后劲

确保投资快速增长。推进79个在建重点项目，30个预备重点项目，33个前期项目，提高重点项目在整个固定资产投资总量中的比重，提高生产性项目在重点项目中的比重。加快福厦铁路莆田段、省道201线莆田段、福建LNG站址、莆田燃气电厂、澄峰围垦工程、林浆纸一体化项目原料林基地、工艺美术城等项目建设，开工建设仙游金钟水利枢纽、抽水蓄能电站、福泉高速公路莆秀支线等项目。做好林浆纸一体化项目跟踪服务，加快立项报批。全力推进向莆铁路、福厦铁路湄洲湾支线、湄洲岛至永春高速公路、沈海线复线莆田段、湄洲跨海桥隧通道、东吴海洋工程等项目前期工作。

千方百计招商引资。以工业园区和工业集中地为载体，围绕电子信息、能源、化工等十大产业集群招商引资。重视吸引欧美资本和跨国公司投资，积极引进中央直属大企业投资。利用“5·18”、“6·18”、“9·8”、莆商大会和妈祖文化旅游节、中国海峡工艺品博览会等招商经贸活动，引进一批高附加值和高科技含量的制造业项目。在抓好工业项目招商的同时，重视对农业、服务业和社会事业领域的招商。积极探索市场化运作的招商新机制，落实招商引资激励政策，提高项目引进成功率。

策划拓展储备项目。注重策划一批效益好、污染少、自主创新能力强的储备项目。做好重点招商项目的编制工作，推动一批促进产业升级的重点项目。培育和挖掘项目源，引导项目开发方向，从发展规划、区位资源优势、民资外资中找项目，从接受产业转移、资源深加工、产品精加工、产业协作配套上找项目，形成新项目不断生成的态势，形成项目滚动发展的持续能力。

（四）加快推进各项改革，以创新激发活力

全面推进各项改革。创新农村工作机制，稳步推进乡镇机构、县乡财政体制和农村义务教育体制改革。做好林权制度改革的配套完善工作。创新政府管理制度，完善工作管理制度和社会评价体系，继续抓好绩效评估和规范统计工作。推进文化体制改革。推行行政执法责任制，规范行政执法行为。贯彻实施《公务员法》，改革公务员工资制度，加快推进事业单位人事制度和分配制度改革。以强化规范财政监管为重点，改革税收征管机制，完善增收节支激励机制，重点解决乡镇财政困难问题，积极化解乡村债务。

加快发展民营经济。继续实施民资回归工程，围绕拓展民营经济发展空间，重点在基础设施、市政公用事业、社会事业以及现代服务业领域推出一批适合民间资本投资的具体项目。继续完善服务工作机制，做好融资担保和技术支持工作，在能源、运输等生产调度上给予扶持。引导民营企业加强管理创新，提升民营企业竞争力，争创全省百强民营企业。

创新外贸促进机制。继续兑现出口奖励办法，重点扶持一批外向型企业，扩大制鞋、电子信息、机电产品、轮胎和鳗鱼等主导产品出口。建立完善外贸出口预警机制，积极应对国际贸易壁垒。着眼于提高服务保障能力，开辟“绿色通道”，提高大通关效率。实施“走出去”战略，引导推动民营企业建立境外生产基地和营销网络，开展对外承包工程和对外劳务合作业务。

（五）推进城市化进程，提升城市带动功能

加快城市发展步伐。注重城市宏观层面规划研究，完善城市规划编制体系，重点抓好“十一五”城市发展专项规划、城市近期建设规划、荔城新区规划、港口岸线规划以及城市重点地段的详细规划，强化规划实施和监督管理。加快编制《湄洲湾港口总体规划》，开工建设LNG10万吨级专用码头、秀屿10万吨级通用码头及5万吨级集装箱码头、4万吨级木材专用码头、5万吨级石油化工码头、湄洲湾10万吨级航道疏浚等6个港航项目，推进东吴港区15万吨级散货码头、林浆纸项目配套一期15万吨级码头等项目前期工作。开工建设荔港大道，向东延伸荔城大道和文献东路，续建荔涵大道涵江段及交战路二期工程、荔园路二期工程、秀屿港城大道等，新增道路面积137万平方米。促进房地产市场和建筑业市场规范有序发展，积极稳妥推进旧城改造项目。

不断完善城市配套设施。加快木兰溪防洪工程（郑坂段）、护城河片区四期改造工程、海堤和城区防洪排涝设施建设，抓好城市防空、防震、防火设施建设，提高城市综合防灾减灾能力。加快市政道路的路灯架设，重点抓好中心城区、荔城大道、文献路和秀屿港城大道等重要路段的夜景工程，配套建设城市管道燃气工程。新建莆田、仙游客运中心站和一批城区停车场、停靠站点，新开、延伸一批城区公交线路。深化城市综合执法改革，落实辖区管理责任制，提高城市管理水平。

统筹县区经济发展。继续落实加快县区经济发展的政策措施，进一步壮大县区经济。发挥荔城、城厢全市政治经济文化中心和交通发达的优势，加快发展工业和服务业，构建现代物流中心，壮大城市经济。发挥涵江侨资、商贸活跃和民营经济起步早的优势，以高新技术园区和商贸批发中心为龙头，加快发展工业和商贸经济，建设工贸城区；用活湄洲湾北岸开发区的优惠政策，发挥秀屿区港口资源优势，大力发展临港工业和海洋经济；发挥仙游县发展县域经济的政策优势和联结沿海与内陆腹地的区位优势，突出发展工业经济、特色农业和旅游经济，做大经济总量；打响湄洲岛国家旅游度假区品牌，建设湄洲岛跨海通道，发展旅游经济和对台交流。

着力改善生态环境。推进垃圾焚烧发电项目，加快建设市污水处理厂二期扩建工程，做好秀屿污水处理工程、仙游污水处理厂等环卫设施项目的前期工作，实施污水、垃圾产业化试点工作。继续实施城乡绿化一体化工程，创建省级园林城市，新建荔浦公园，续建东岩山公园、延寿公园和兰溪南岸公园，开发凤凰山沿渠、木兰溪下游两条景观带。加强水源生态环境保护，做好东圳库区水土保持生态工程和木兰溪、萩芦溪水污染综合整治，推进“青山挂白”整治和城乡生态林、防护林建设。积极推进资源节约，做好节水型社会建设试点工作。

（六）加大社会事业建设，促进协调发展

加快推进科教进步。继续实施科教兴市战略，构建创新型莆田，鼓励全社会提高原始创新能力、集成创新能力和引进消化吸收再创新能力。制定支持企业建立技术研发中心的办法，鼓励企业加强创新能力建设。推动高校、科研单位和企业共建产学研经济实体及面向全行业的技术开发，加快共性技术和关键技术开发。建立常设技术市场，推进科技中心项目前期工作。重视幼儿教育，办好中心幼儿园。巩固“两基”成果，推进“双高普九”工作，新增省高中示范校2所、省级达标校5所，加快发展职业教育，提升高等教育办学水平。加大对农村教育的支持力度，在师资配备和教学设施建设上重点向农村倾斜。继续抓好第二期危房改造，确保52个危改项目投入使用。鼓励社会力量办学。推进莆田学院和湄洲湾职业技术学院建设。

大力加强公共卫生。加强对传染病、职业病监测预防和精神病、心理疾病防治，严格防范禽流感等动物疫病。加快建设莆田人民医院、福兴医院、莆田学院附属医院新区工程和市第一医院惠妹外科医技大楼，配套建设市急救中心、第五医院、疾控中心，争取年内投入使用。大力发展社区卫生服务，加强农村初级卫生保健工作，在做好荔城区新型农村合作医疗试点工作的基础上，扩大到城厢、涵江两区。

丰富发展城乡文化。鼓励城市科技、教育、文化、卫生等机构和人员到农村开展服务。保护莆仙戏等民间文化和文物，加快莆仙大剧院建设。推进基层文化阵地建设，支持和推进县区文化馆、图书馆建设。筹建福建妈祖乐府，开展对外特色文化交流。发挥市广电中心作用，推进广播电视“村村通”工程。完善市体育中心等体育设施，积极申办第十四届省运会。继续落实计划生育基本国策，稳定低生育水平。同时，做好档案、地方志等工作。

（七）保障人民群众利益，建设和谐莆田

完善就业和社会保障制度。落实国务院新一轮就业再就业政策，建立健全城乡一体化就业服务体系。发展技工教育，加强职业技能培训。健全再就业援助制度，重点做好大中专毕业生就业工作。建立解决拖欠农民工工资和建设领域拖欠工程款问题的长效机制，尽快制定被征地农民基本生活保障的办法。继续扩大社会保险覆盖面，健全多层次的医疗保障体系，完善社会救助体系，开展农村养老保险试点工作。成立莆田市慈善总会，加快发展福利事业。积极发展残疾人事业，加强老龄工作，建设老年文化教育中心。

推进平安和谐莆田建设。深入开展“平安莆田”创建活动，全面实施“五五”普法规划，加强社会治安综合治理工作，严厉打击各类违法犯罪活动。全面落实安全生产责任制，开展安全生产专项整治，防范和减少重特大安全生产事故的发生。进一步完善信访工作责任制，引导群众以理性合法的方式表达利益诉求。建立和完善各类预警和应急处置体系。推进诚信社会建设，整顿市场经济秩序。深入开展双拥共建活动，积极创建新一轮“全国双拥模范城”。

办好惠民实事。落实好省政府办好惠民实事的承诺。落实城乡居民最低生活保障制度，落实农村“五保”供养政策，全面实施农村义务教育免除学杂费工作，继续实行农村低保家庭子女接受义务教育免费提供课本和寄宿生生活补助制度。完善农村供水设施，加快建设南日岛跨海供水工程。健全救灾工作体制，完善各级救灾应急预案和救灾物资储备系统。实施食品、药品放心工程，治理餐桌污染，纠正医药购销和医疗服务中的不正之风。

四、提高效率　建设对人民负责的政府

效率关系事业，效率决定成败。提高政府工作效率，是建设对人民负责政府的基本条件，是转变职能、服务发展的必然要求，是经济翻番、港城崛起的重要保证。各级政府要抓住换届选举的契机，以强烈的发展意识、宽广的世界眼光、求真务实的精神、攻坚克难的魄力、开拓创新的冲劲、服务大局的合力，加快推进湄洲湾港口城市建设。

提高效率，要体现在推动发展上。推动发展既是实现效率的过程，也是检验效率的标准。在推动发展过程中，要重视抓好细节，细节反映效率，细节牵动全局，通过分解目标、细化任务，推动每一个项目的落实、每一项任务的完成、每一项措施的实施，确保政府工作预期目标的实现。要注重解决问题，把经济发展的难点、群众关心的热点作为解决问题的重点，强调主动解决问题的态度，提高解决问题的能力，把问题解决在一线、把问题解决在第一时间。要抓好工作落实，建立健全抓落实的长效机制，明确岗位责任和目标要求，凡是确定的事情，特别是事关发展大局的重点工程、重大项目、重要事项，做到有布置、有跟踪、有督查、有考核，推动目标落实、责任落实、工作落实。

提高效率，要体现在开拓创新上。创新出效率。要创新发展思路，面对战略转型的新要求，必须进一步解放思想，更新观念，汲取先进地区的经验，制定新一轮发展战略，在创新发展模式、转变经济增长方式上有所作为。要创新体制机制，推进行政许可和非行政许可审批制度改革，创新政府运行机制和管理方式，坚决改变一切束缚发展的做法和规定，以体制机制创新保障高效运转。要创新工作方法，把工作着力点放在为市场主体服务、为企业服务上，善于运用法律的手段、市场的机制、发展的办法解决发展中存在的困难和问题，创造性地开展工作，力求更快更好地发展。创新源自学习，要建设学习型政府，勤于学习、善于学习，不断提高创新能力，使政府工作更好地体现时代性，把握规律性，富有创造性。

提高效率，要体现在改进作风上。提高政府效率，改进作风是保证。改进作风必须强化大局意识，自觉在推进湄洲湾港口城市建设大局中找准位置、主动融入，在参与发展、服务发展、推动发展中发挥作用、展示水平、体现价值。改进作风必须强化责任意识，责任决定效率，责任成就事业，严格落实工作责任制和责任追究制度，该负的责任勇于承担，该管的事主动管，该解决的问题及时解决，一切以效率为先。今年还是“项目工作攻坚年”，要进一步落实责任，加强重点项目任务的分解和跟踪管理。进一步增强合力，在项目报批、征地拆迁、供水供电等方面加强协调配合，为重点项目创造良好的建设环境。严格落实项目业主责任制，抓好质量、进度、安全监控，确保按计划实施。改进作风必须坚持求真务实，克服官僚主义、形式主义，纠正文牍主义，简化繁文缛节，开解决问题的会，发有实际内容的文，倡导简政、高效、务实的作风。

提高效率，要体现在接受监督上。监督是提高效率的外在动力。政府的一切权力来自人民，必须对人民负责，主动接受监督。各级政府要自觉接受同级人民代表大会及其常委会的监督，接受人民政协的民主监督，认真听取民主党派、工商联、无党派人士和各人民团体的意见，建立邀请人大代表、政协委员和群众代表参与政府重大决策的制度。接受新闻舆论和社会公众监督，健全重大决策公示制度和听证制度，建立政府新闻发言人制度。加强政府系统内部监督，支持监察、审计部门依法独立履行监督职责。推进机关效能建设，提高政府部门及其工作人员的服务效率和质量。

提高效率，要体现在勤政廉政上。坚持从严治政、廉洁从政，全面落实党风廉政建设责任制，落实“五条禁令”和四项制度。建立健全教育、制度、监督并重的惩治和预防腐败体系，努力从源头上预防和治理腐败。大力弘扬艰苦奋斗、勤俭节约的优良传统，坚决遏制铺张浪费、奢侈攀比之风。进一步完善政务公开制度，增强政府工作的透明度。继续深入开展专项治理，纠正部门和行业的不正之风。加强廉洁自律教育，建设一支政治上靠得住、工作上有本事、作风上过得硬的公务员队伍。

各位代表，宏伟目标的实现需要长期努力和合力攻坚。让我们紧密团结在以胡锦涛同志为总书记的党中央周围，在省委、省政府和市委的领导下，团结和依靠全市人民，把握新机遇，迎接新挑战，创造新业绩，为加快建设湄洲湾港口城市而努力奋斗！
